# Factors Affecting Staff Turnover of Young Academics: Job Embeddedness and Creative Work Performance in Higher Academic Institutions

**DOI:** 10.3389/fpsyg.2020.570345

**Published:** 2020-12-14

**Authors:** Imran Ahmed Shah, Amit Yadav, Farman Afzal, Syed Maqsood Zia Ahmed Shah, Danish Junaid, Sami Azam, Mirjam Jonkman, Friso De Boer, Ronju Ahammad, Bharanidharan Shanmugam

**Affiliations:** ^1^School of Management and Economics, University of Electronic Science and Technology of China, Chengdu, China; ^2^Department of Information and Software Engineering, Chengdu Neusoft University, Chengdu, China; ^3^Institute of Business and Management, University of Engineering and Technology, Lahore, Pakistan; ^4^Department of Statistics, Shah Abdul Latif University, Khairpur, Pakistan; ^5^College of Engineering, IT & Environment, Charles Darwin University, Darwin, NT, Australia

**Keywords:** organizational embeddedness, community embeddedness, young teachers, creative work performance, voluntary turnover intentions

## Abstract

Young academics have been facing a problem of high turnover rate due to missing links between the institutions’ policies and the performance. This study explores the effect of job embeddedness and community embeddedness on creative work performance and intentions to leave of young teaching staff in academic institutions in Pakistan. In this study, 300 qualified young academics from public and private universities were selected as subjects and asked to complete a questionnaire. Data were collected via mail-survey. A variance-based structural equation model is employed to measure the path model. The results show that the fit-dimension of organizational- and community-embeddedness, along with the moderating effect of organization size and the availability of nearby alternative jobs have a significant impact on improving perceived creative performance and reducing staff turnover intentions. This study suggests that organizations should focus on organizational-fit and community-fit constructs in their nurturing strategies to embed young teachers in their academic institutions. This study also suggests that monetary rewards only are relatively ineffective to improve retention. Hence, public and private sector universities should facilitate meaningful contributions from young teachers in creative work and provide opportunities for social interactions and personal development.

## Introduction

Retaining talented faculty members is important for universities to enhance the institutions capacity in response to the changing dynamics of research and higher education globally (Coetzer et al., 2018). But recently, attracting and retaining talented young teaching staff has become progressively challenging as a result of changing social, economic, demographic, and psychological trends (Dechawatanapaisal, 2018a). A high turnover rate of teachers can be costly for academic institutions. Institutions may have to invest in resource building (i.e., offering salary benefits) in order to retain their teaching staff (Akgunduz and Eryilmaz, 2018). Otherwise, the institutions might need to enter in the hiring process repeatedly to attract the best talent available which results in high costs (Thakur and Bhatnagar, 2017).

Job embeddedness (JE) theory emphases on the key decision-factors that explain why employees are willing to stay (Allen et al., 2016). These decision-factors (i.e., link, fit and sacrifice) can affect the retention of staff as well as employee performance. In contrast to some traditional approaches of addressing staff turnover that focuses more on job satisfaction (Zimmerman and Darnold, 2009), JE suggests that the personal life of a person influences the decision to stay in the present job.

Job embeddedness theory predicts that greater perceived job security through organizational- and community- would lead to an increase the employees’ creative work performance (CWP) within the organization and a decrease in their propensity to look for alternative jobs (Karatepe and Vatankhah, 2014; Rahimnia et al., 2019). However, a better thoughtful of the correlation between JE, CWP, and voluntary turnover intention (VTOI) is required to better address the challenging questions of staff performance and retention (Karatepe and Vatankhah, 2014).

Recently some studies have investigated the role of JE toward turnover and performance in the services sector, particularly in the health sector (Chang et al., 2018). Limited studies have been conducted concerning the importance of JE for academic institutions (Mitchell et al., 2001; Dechawatanapaisal, 2018b; Rahimnia et al., 2019). Currently, a substantial proportion of the variability in performance and turnover in higher academic institutions remains unexplained (Shahid, 2018). Higher academic institutions are hubs of the career development of young people (Yang et al., 2019). Young faculty is often struggling to get essential organizational support which may help them to improve their performance (Faberman et al., 2017). Currently, the tendency to look for other jobs among young faculty members in countries such as Pakistan is very high. This is due to several factors, such as a focus on working in metropolitan cities, financial benefits, job security, and the need for professional development opportunities. Achieving a high work performance is a big challenge globally with the continuous pressure of ranking of academic and research outcomes (Ghaffar and Khan, 2017).

In recent times, the need to address the faculty responsibilities and working conditions have become more urgent. When experienced and competent teachers show VTOI, productivity in terms of academic performance tends to decrease (Chen and Ayoun, 2019). According to the JE theory, it is essential to address decision-factors that lead toward low JE and negative contributions to organizational and personal performance thereby results in early turnover (Ferreira et al., 2017). To date, no empirical studies have sufficiently explained these embeddedness-related issues on CWP and VTOI among young teachers. A better understanding of these issues might lead to more effective practices related to the organizational embeddedness (On-the-JE) and community embeddedness (Off-the-JE) for academic staff. Considering that this research aimed to explore the effect of JE on CWP and intentions to leave of young teaching staff in Pakistan. Subsequently, this study addresses the following research question: How does on-the-job embeddedness and off-the-job embeddedness increase the level of CWP and reduce VTOI among the faculty members of higher academic institutions? Subsequently, we try to identify effective organizational practices that are likely to enhance the JE among faculty members. In addition, it is essential to find which variables could moderate the effect of JE on VTOI. This study may help to explore the important antecedents of JE and viable strategies that could enhance the embeddedness among faculty.

The findings of this study demonstrate different significant implications for organizations looking for developing effective retention strategies. The results of this study provide useful, practical implications for organizations and managers seeking to develop effective retention strategies actively particularly in academics. Specifically, this study suggests that institutions should focus on organizational-fit and community-fit constructs in their nurturing strategies to embed young teachers in their academic institutions in the future. Subsequently, this study also suggests that monetary rewards are only comparatively trivial to the retention practices.

This paper proceeds with an explanation of the theoretical background of JE issues among faculty of higher academic institutions resulting in the development of a research hypothesis in section “Theoretical Background and Hypothesis Development” and a description of the research design and the procedural steps taken for data collection in the section “Materials and Methods.” The section “Data Analysis and Results” consists of a detailed analysis of the proposed framework and hypothesis testing and section “Study Findings and Discussions and Implications” presents the key findings and the proposed managerial practices for strong embeddedness among faculty. Finally, in the section “Conclusion,” the conclusion, limitations, and future direction of the study are discussed.

## Theoretical Background and Hypothesis Development

Previous literature (Wheeler et al., 2012; Karatepe and Avci, 2019) explained the role of JE theory in order to address the turnover and performance-related problems in different types of organizations including manufacturing and the service sector. The following section describes the theoretical background of JE and how it relates to the turnover and performance-related problems among teaching staff.

### Job Embeddedness (JE) and Voluntary Turnover Intention (VTOI)

Job embeddedness refers to a state of mind where an employee would decide to stay with an organization. This is due to certain organizational-related decision-factors (Robinson et al., 2014). These decision-factors are related to on-the-job (organizational) or off-the-job (community) embeddedness and affect both the employee’s performance and turnover (Peachey et al., 2014). JE can be measured through three different constructs called links, fit and sacrifice. Fit is defined as the compatibility of an employee with the current organizational culture and the surrounding community. Links refer to the employee’s connections within the organization or with an external community that may influence the decision process. Sacrifice describes the level of opportunity cost for the employee when leaving the job (Reitz, 2014).

In the past, researchers have applied the traditional model of Ampofo et al. (2017) in which displeasure was seen as the primary source of low performance and high turnover (Choi and Kim, 2015; Hussain and Deery, 2018). Later, Mitchell et al. (2001) have investigated key dimensions of JE which are linked with VTOI beyond the obvious factors, such as job satisfaction and commitment. Holtom and Inderrieden (2006) have presented a model of JE describing a unique perspective to the question: “Why do people stay or leave the job?” Over the years, several researchers have worked on the relations between embeddedness, performance and turnover. Their empirical findings suggested that JE characteristics predict both the employee’s intention to quit and actual turnover (Choi and Kim, 2015; Ampofo et al., 2017). Bergiel et al. (2009) established the significance of JE as a link amongst organizational practices and purpose to leave. They argued that if organizational practices are effective in embedding their employees, then they would slow turnover. Meta-analytics of data of various studies also suggest that JE has a reliable negative impact on turnover intention (Jiang et al., 2012). Several other recent studies have established that improved JE tends to reduce VTOI in organizations (Reitz, 2014; Chan et al., 2019).

Similarly, organizational-and community-embeddedness affect the decisions related to VTOI in academics (Dechawatanapaisal, 2018b). Recently, a study conducted in the Iranian education sector revealed that organizational practices sometimes have an adverse effect on turnover if teachers perceive organizational support is low. Based on above discussion, it is pertinent to study deeply about JE characteristics in turnover theory. Limited studies (Rahimnia et al., 2019) have been found related to turnover intention that particularly emphasis on the different dimensions of JE on VTOI that enhance the organizational support in the form of better embeddedness, a long with the role of CWP. Therefore, it is pertinent to have deep routes of JE characteristics to extend the turnover theory in education sector, where creative role performance in the form of teaching and research in important. Table 1 describes the basic definition of fit, link, and sacrifice in terms of organizational and community roles.

**TABLE 1:** Job embeddedness dimensions for young teachers.

JE dimension	Organization	Community
Fit	Organizational fit: A young teacher’s perceived compatibility with the institution.	Community fit: How well a young teacher perceives that he/she fits in the surrounding community and environment.
Link	Organizational links: Formal or informal connections that exist between a young teacher and co-workers in the university.	Community links: The significant influence of family and other social institutions and their influence on decision making.
Sacrifice	Organizational sacrifice: What a young teacher would have to give up to break free from links from the university.	Community sacrifice: The ease that links can be broken between the teacher and the surrounding environment.

#### Organizational Embeddedness (On-the-JE) and VTOI

Young teaching staff become more embedded where there is a good fit with their career goals and opportunities within the organization (Chen et al., 2018). It is observed that organizations that gives opportunities to young teachers for achieving their career goal tend to show results in more embeddedness (Watson, 2018). The higher the perceived organizational support, the higher the tendency being attached to the organization (Dechawatanapaisal, 2018a). Interpersonal relationships of young teachers with co-workers are required for a good working environment (Hussain and Deery, 2018). Finally, there are some other factors that make leaving a job more difficult such as the sacrifice in terms of giving up valuable perks or benefits (Vardaman et al., 2018). Therefore, the hypothesis related to effect of the on-the-job characteristics (i.e., link, fit, sacrifice) of JE on VTOI is as follows:

H1a: The effective implementation of organizational embeddedness (On-the-JE) practices can reduce the voluntary turnover intention (VTOI) of young faculty in higher academic institutions.

#### Community Embeddedness (Off-the-JE) and VTOI

Similar to the organizational-embeddedness, it has also become apparent that community-embeddedness, particularly in more remote areas, is also an important aspect of JE which may have an impact on turnover (Yang et al., 2018). Social networks can help academic teaching staff to adjust in their work environment (Shehawy et al., 2018). If the employee’s community links are weak, VTOI might be higher. On the other hand, if the community networks are strong then the perceived costs of leaving the current job would increase (Holtom et al., 2019). There are some evidences showing that if the perception of leaving the community is difficult then it is more difficult to quit a current job (Dechawatanapaisal, 2018b). Therefore, this study strongly argued that community-embeddedness is linked with the VTOI among young teachers. If organizations work effectively on community-related factors and provide the necessary support, then young teachers can establish social networks resulting in low VTOI. Therefore, it is hypothesized here as follows:

H1b: The effective implementation of community embeddedness (Off-the-JE) practices can reduce the voluntary turnover intention of young faculty in higher academic institutions.

### Job Embeddedness (JE) and Creative Work Performance (CWP)

Creativity plays an important role in education and teaching, in a way of knowledge creation and in sharing (Wang and Netemeyer, 2004). Creativity is the process of introducing new knowledge or ideas and may result in a positive change in organizational norms (Wang and Netemeyer, 2004). CWP can be explained as the capability to generate new solutions for effectually solving problems and the behavioral propensity of individuals to be imaginative at the workplace. It can be linked with the performance of academics when they can create new ideas for teaching and research (Rahimnia et al., 2019).

High CWP at the workplace is related with higher on-the-job or off-the-job organizational support. Therefore, JE theory can also address the significance of CWP for teaching staff. Rendering to this theory, teaching staff with a high level of JE tend to introduce innovative methods for teaching and demonstrate a higher involvement in their teaching responsibilities. In addition, higher organizational support in terms of embeddedness would also encourage teachers to share their knowledge. Consequently, teachers achieve higher levels of creativity levels in their roles (Karatepe and Avci, 2019). Academics may also be aware that low CWP in their early careers can result of the loss of benefits at a later stage (Rubenstein et al., 2019).

#### Organizational Embeddedness and CWP

The relationship between On-the-JE and CWP can also be explained with the state of “fit” in JE theory (Chen and Ayoun, 2019). When teachers feel that they are embedded in their job, then they show improved performance at the workplace (Hobfoll, 2001). Subsequently, embedded teachers who feel that there is a decent fit with the job and organizational culture may have increased motivation toward CWP (Karatepe and Avci, 2019). Since organizational embeddedness makes a great number of links, a sensation of compatibility with their work can make a teacher more motivated to stay and execute in a more creative manner. Therefore, embedded young teachers who are more content with their working environment and its benefits tend to perform better, and make more use of their creative abilities. It is hypothesized here that:

H2a: The effective implementation of organizational embeddedness (On-the-JE) practices can enhance creative work performance (CWP) of young faculty in higher academic institutions.

#### Community Embeddedness and CWP

Evidence showing the community-related factors increase CWP at the workplace has been described by Wheeler et al. (2012). Teaching staff that are well embedded in their community and social networks have good relations with family and friends. In addition, they may also participate in healthy social activities (Mitchell et al., 2001). That community-embedded teacher may perform better in their job because they feel that organizational and community-related support provides them with the opportunities to fit in a social network. Thus, CWP can be enhanced by continuous community-embeddedness practices. If a teacher has a good social and private life, he/she may also perform better in the university. Based on the arguments above, it is hypothesized that:

H2b: The effective implementation of community embeddedness (Off-the-JE) practices can enhance creative work performance (CWP) of young faculty in higher academic institutions.

### CWP and Voluntary Turnover Intention (VTOI)

As discussed earlier, CWP has an effect on the teacher’s VTOI (Swider et al., 2011). Karatepe and Avci (2019) have studied work performance and found that employees’ involvement in work correlates negatively with turnover intention. The performance level seems to directly affect the decision of teachers to stay or look for alternative jobs. High-performing academics seem to be more satisfied with organizational policies and the available benefits and tend to stay on the job for longer periods of time than low-performing academics (Ghaffar and Khan, 2017). The study finds that the tendency of CWP is linked to increased expectations of better rewards in the future. If a teacher has a high tendency of CWP, they are less likely to quit a job. Similarly, low-CWP and less perceived organizational support tends to lead increase in anxiety, frustration, and a higher tendency to leave the organization (Yıldız, 2018). Therefore, it has been hypothesized that if teachers are involved in creative work activities they will be more satisfied and tend to show lower VTOI.

H3: creative work performance (CWP) is negatively related to Voluntary Turnover Intention (VTOI).

### Possible Mediation of CWP

Staying in an organization depends on the performance of an employee. Usually, the employees who perform better tend to stay for a longer period of time and show a lower VTOI. Thus, if young teachers are performing well and the organization is also supporting them in their creative role performance, they might prefer to stay in their job rather than showing early turnover intentions. Earlier, it has been argued that JE can have a major effect on role performance and creative work. Therefore, CWP can play a mediating role between JE practices and the teacher’s VTOI. Various studies describe this causal relationship between JE practices and VTOI in relation to the perceived CWP in the organization (Hussain and Deery, 2018; Holtom et al., 2019). Therefore, it is hypothesized here:

H4a and b: Creative work performance (CWP) mediates the relationship between organizational embeddedness (On-the-JE), community embeddedness (Off-the-JE) and voluntary turnover intention (VTOI), such that this relationship is weaker if creative work performance is high.

### Possible Moderators

Researchers have argued that different JE-related turnover models require the addition of some potential (Choi and Kim, 2015).

#### Organizational Size

Academics in large organizations are likely to be more satisfied with the opportunities for professional growth and advancement within their organizations. Young academics in prominent universities may be more likely to receive opportunities for training and development than teachers in small universities (Young, 2012). Furthermore, large universities are more likely to have career succession plans that may encourage long term commitment of staff. Teachers in public (large) and private (small) universities may have different perceptions related to organizational fit, link and sacrifice, thereby teachers in large universities (300 teachers) have better support than teachers in small universities (50 teachers). Based on this argument, this study poses the following hypothesis:

H5a and b: Organizational size moderates the relationship between organizational embeddedness (On-the-JE), community embeddedness (Off-the-JE) and turnover intentions, such that this relationship is weaker if the organizational size is small.

#### Availability of Nearby Replacement Jobs

The availability of potential replacement jobs nearby may also moderate the relationship between organizational-/community-embeddedness and turnover, in such a way that despite the effective embeddedness practices, a turnover rate may be increased if alternatives job opportunities are available at nearby locations. In other instances, finding a new job might require someone to travel long distance from the community in which they are currently living (Wijayanto and Kismono, 2004). If a university teacher has a great community attachment, then it may be challenging to quit his or her previous job. This study intends to test whether the perception of the availability of nearby replacement jobs moderates the relationship between organizational-/community-embeddedness and turnover. Therefore, it is hypothesized as follows:

H6a and b: The perception of the availability of nearby replacement jobs moderates the negative relationship between organizational embeddedness (On-the-JE), community embeddedness (Off-the-JE) and turnover, such that relationship is weaker if the availability of nearby replacement jobs is high.

### Theoretical Framework

**FIGURE 1:**
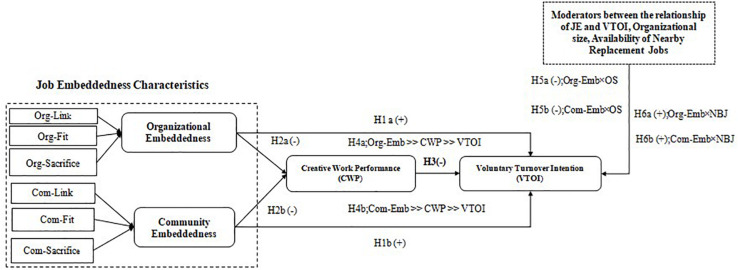
Theoretical framework.

Based on the theoretical background and above discussion for hypothesis development, this study proposes a novel framework for understanding the role of JE in determining CWP and VTOI. In addition, different moderating effects that could impact on the relationships are also measured. Figure 1 shows a relationship between key JE domains, CWP and VTOI along with the important moderators.

The main characteristics of JE within the organization and employees-related community can be divided into three groups: fit, links, and sacrifice. As discussed earlier, fit is the compatibility of an employee with the current organizational culture and the surrounding community. Links refers to the employee’s connections within the organization or with the external community that may influence the decision process. Sacrifice describes the level of opportunity cost that an employee has when leaving the jobs.

## Materials and Methods

A positivist paradigm of research design is adopted for this study because its epistemological positioning allows researchers a legitimate way of primary data generation, by interacting with people, asking questions and recording responses through standard questionnaires (Ampofo et al., 2017).

### Context

To test the conceptual model, the data is collected, through a mail survey, targeted toward young faculty members of different public and private universities in Pakistan. Academic faculty contributes to the development not only of students but also of society in general and faculty retention is an important issue in many universities in Pakistan. Nowadays, the education sector in Pakistan is an emerging market for private sector investment. Also, university teachers are being considered the best educated members of the community, but they have been facing serious issues of JE in this rising sector. As a result, they feel that they are not able to perform well and are thinking about job switching. Many academics consider migrating abroad in order to obtain better skills and improve their future, because of the lack of development programs available for teaching staff in their own university. This study intends to investigate the critical factors of JE resulting in low performance and high turnover among young teaching staff in Pakistan.

### Instruments

The JE instrument developed by Mitchell et al. (2001) was used to assess the level of JE among young academics. The JE instrument is based on a forty items tool along with a seven-point Likert-scale and is designed to measure JE in the education sector (Thakur and Bhatnagar, 2017; Dechawatanapaisal, 2018a; Costantini et al., 2019; Holtschlag et al., 2019; see Annexure 1). The JE instrument is grouped into organizational and community related sub-scales. Within each group, there is a subscale according to link, fit and sacrifice such as organizational fit, community fit, organizational links, community links, organizational sacrifice and community sacrifice.

Similarly, a list of items pertaining to CWP (Greene et al., 2018; Khan et al., 2018) and VTOI (Choi and Kim, 2015; Hussain and Deery, 2018) was also derived from the extant literature. Whereas VTOI consists of three measurement items, CWP consists of four measurement items. Annexure 1 also describes the measurement items of VTOI and CWP, respectively. In addition, two potential moderators, organizational size and availability of a nearby replacement jobs are measured as they have been shown in literature to have a moderating effect on VTOI. Organizational size is measured on a scale of Small or Big. Similarly, the availability of a nearby job is measured on a binary scale of “Yes” or “No.”

In this study, we selected 30 respondents from the population of young teaching staff or pre-testing of the instrument. In order to ensure the content and face validity of the questionnaire, the young teachers were briefed on the purpose of this research. Data on demographic and organizational characteristics including gender, age, and job-status, were also collected as can be seen in Table 2.

**TABLE 2:** Characteristics of sample (*N* = 300).

Characteristics	Classifications	Frequency	Percentage (%)
Gender	Male	215	71.67
	Female	75	28.33
Age	Below 30 years	41	13.67
	31–40 years	175	58.33
	above 40 years	84	28.00
Type	Public	195	65.00
	Private	105	35.00
Area	Sindh	145	48.33
	Punjab	108	36.00
	KPK	25	8.33
	Baluchistan	22	7.33
Job-designation	Lecturer	65	21.67
	Assistant professor	152	50.67
	Associate professor	83	27.67
Job-status	Permanent	94	31.33
	Contract	138	46.00
	TTS	68	22.67

### Sample and Procedure

The inclusion criteria of this data collection included young teaching staff having a Ph.D. degree and working as a permanent faculty member with research and teaching tasks in a university in Pakistan. The majority of the participants are working as a lecturer, assistant professor, and associate professor in public and private universities across Pakistan. A study sample has been collected from different provinces of Pakistan in a way to measure embeddedness differences in different regions with different educational policies. In addition, as a result of the male/female ratio in these positions, this study includes a larger number of males then the females.

The initial power analysis for determining the minimum sample size required for this study indicated that 300 participants were needed for the six variables (i.e., On-the-JE, Off-the-JE, CWP, VTOI, organizational size and availability of nearby replacement jobs) (see Table 2). A list of teaching staff working at public and private universities was obtained from the higher education commission of Pakistan. Potential participants were initially grouped and then randomly chosen from this list. A packet consisting of a cover letter and a questionnaire was sent to each potential participant with an identification number to differentiate respondents from non-respondents. A reminder memo was later sent to the non-respondents. A total of 375 survey packets were sent out in three successive mailings resulting in an 80% response rate. Twenty-nine of the participants did not meet the inclusion criteria of the study, thereby leaving a total of 269 useable surveys for further analysis.

## Data Analysis and Results

In view of testing the hypothetical model, structural equation modeling (SEM) has been employed to assess the direct, indirect, total and interaction effects of the variables. Considering the sensitivity of sample size and other psychometric properties of scale, a variance-based SEM approach has been adopted.

### Reliability and Validity Measurement

In order to assess the psychometric properties of the scales being used, reliability was measured using composite reliability (ρ) and internal consistency (α) whereas, validity was measured using convergent and discriminate validity (Watson et al., 1988; Cable and DeRue, 2002). Factor loadings and α of each latent variable show that the conformity of scale holds true for path measurement (see Table 2). For the scale validity, α and ρ scores are found to be greater than 0.7, confirming scale reliability (see Table 3).

**TABLE 3:** Factor loadings scale summary.

Constructs	Items	Loadings	α	Kurtosis	Z-Ratio	KMO
Creative work performance	CWP1	0.8707	0.895	–0.541	–0.298	0.801
	CWP2	0.861	0.882			
	CWP3	0.7515	0.823			
	CWP4	0.8592	0.854			
Voluntary turnover intention	VTOI1	0.8809	0.933	1.196	0.978	0.722
	VTOI2	0.8753	0.921			
	VTOI3	0.878	0.904			
Community embeddedness	Comfit	0.923	0.855	–0.125	–0.445	0.836
	Com-link	0.867	0.752			
	Com-sac	0.841	0.735			
Organizational embeddedness	Org-fit	0.909	0.872	1.585	1.217	0.735
	Org-link	0.863	0.741			
	Org-sac	0.812	0.806			

*CWP, creative work performance; VTOI, voluntary turnover intention; Org-fit, organizational fit; org-link, organizational sacrifice; com-link, community link; com-fit, community fit; com-sac, community sacrifice; KMO, Kaiser-Meyer-Olkin.*

Convergent validity refers to the extent to which the test of the scores correlates with the scores of the other tests that are intended to assess the same construct. In the convergent validity, the minimum value of the AVE should be ≥0.5. Thus, Table 4 shows that the AVE values of all the variables hold the true nature of convergent validity. Similarly, to establish discriminant validity, the value of the square root of AVE for each variable is supposed to be higher than the shared variance among variables, as describes in Table 4. Thus, the values of the square root of each variable are higher than the correlation among variables. In addition, the scores of cross-loadings also show that discriminant validity holds true. Table 4 describes the convergent and discriminant validity of all observed variables that show that the validity holds true for all variables.

**TABLE 4:** Convergent and discriminant validity of all observed variables.

Constructs	AVE	ρ	SQ of AVE
Comfit	0.651	0.834	0.807
Com-link	0.741	0.851	0.861
Com-sac	0.683	0.811	0.826
Org-fit	0.633	0.873	0.796
Org-link	0.752	0.831	0.867
Org-sac	0.695	0.799	0.834
CWP1	0.686	0.816	0.828
CWP2	0.766	0.896	0.875
CWP3	0.721	0.850	0.849
CWP4	0.738	0.788	0.859
VTOI1	0.670	0.769	0.819
VTOI2	0.693	0.860	0.832
VTOI3	0.682	0.811	0.825

### Measurement Model Testing

**FIGURE 2:**
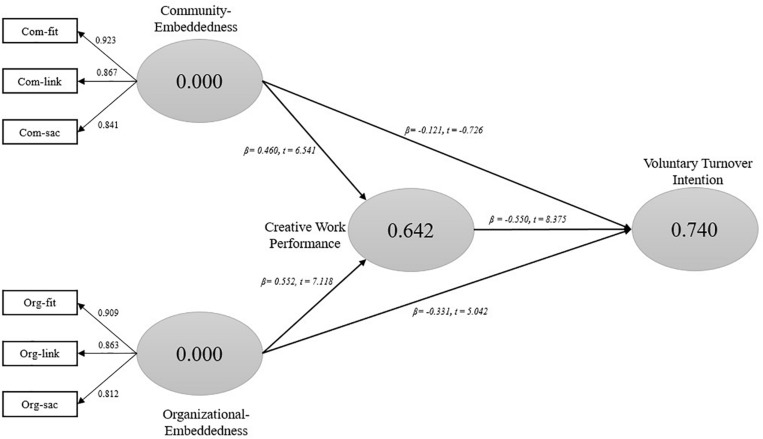
Path model results.

In order to run the SEM for hypothetical path analysis in a model, this study has employed a bootstrapping technique. This technique helps in calculating the path estimates and corresponding *t*- and *p*-values. Figure 2 shows path modeling of the conceptual framework. Direct, indirect, and total effects of various relationships in the theoretical model, along with *t*-, *p*-values, and confidence intervals, are presented in Table 5.

**TABLE 5:** Path model results.

Hypothesis	Relationships	Std. estimated	*t*-Value	BCI		*p*-Value	Results
	
				Lower	Upper		
**Direct effects**							
H1a	Org-Emd → CWP	0.522	7.118	0.267	0.442	0.000***	Supported
H1b	Com-Emd → CWP	0.460	6.541	0.183	0.381	0.000***	Supported
H2a	Org-Emd → VTOI	–0.331	5.042	0.529	0.663	0.000***	Supported
H2b	Com-Emd → VTOI	–0.121	–0.726	–0.147	0.331	0.173	Not-supported
H4a	CWP → VTOI	–0.551	8.375	–0.529	–0.663	0.000***	Supported
–	OS → VTOI	0.354	6.543	0.229	0.436	0.000***	Supported
–	NBJ → VTOI	0.283	3.381	0.197	0.361	0.000***	Supported
**Indirect effects**							
–	Org-Emd → VTOI	–0.375	5.152	0.122	0.288	0.000***	Supported
–	Com-Emd → VTOI	–0.617	8.452	0.204	0.339	0.000***	Supported
**Total effects**							
H3a	Org-Emd → CWP → VTOI	–0.413	6.214	0.129	0.288	0.000***	Supported
H3b	Com-Emd → CWP → VTOI	–0.581	7.747	0.113	0.347	0.000***	Supported

***p < 0.01, BCI = Bootstrap Confidence intervals.

The results indicate that Com-Emd positively influences organizational CWP (β = 0.460, *p* < 0.000); thus, H1b is accepted. Similarly, the relationship between Com-Emd and VTOI (β = −0.121, *p* < 0.173) is also negative but insignificant as par, thus, H2b is not supported. The direct effects of Org-Emd on CWP (β = 0.522, *p* < 0.000) and VTOI (β = −0.331, *p* < 0.000) were significant showing a negative relationship with VTOI, therefore, H1a and H2a are accepted. Two control variables (OS, NBJ) also show a significant relationship with indirect effect results (see Table 5). In the case of indirect effects and total effects between Org-Emd, Com-Emd, and VTOI, all the hypothetical relationships are accepted with a significant negative relationship in path modeling results.

### Moderating Effect

**FIGURE 3:**
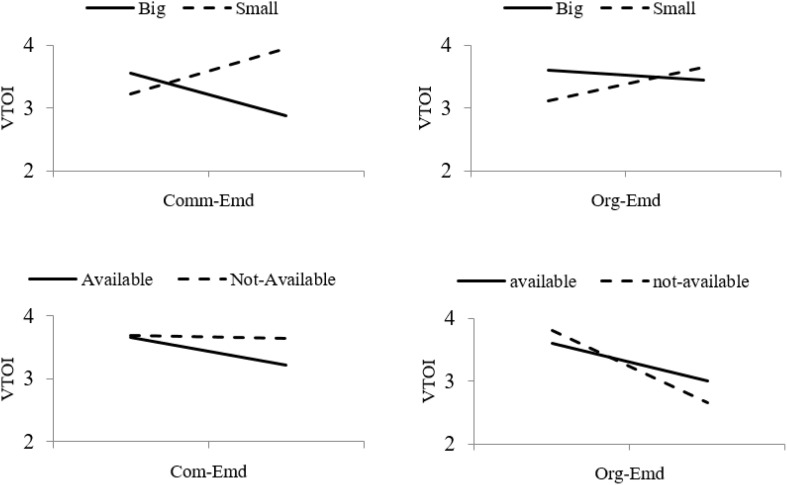
Moderating effects of organizational size and availability of nearby replacement jobs on the relationship between Org-Embeddedness, Com- Embeddedness and voluntary turnover intention.

Apart from the latent constructs, two additional variables, i.e., organization size (OS) and availability of nearby replacement jobs (NBJ), are included in the conceptual model to control their confounding effects. The results indicate significant impacts of OS and NBJ on VTOI. All moderating effects are showing significant effects. However, a moderating effect of NBJ between Com-Emd and VTOI shows an insignificant impact (β = −0.087, *p* = 3.118). Figure 3 and Table 6 describe a brief understanding of the moderating effects of OS and NBJ on Org-Emd, Com-Emd, and VTOI relationships.

**TABLE 6:** Moderating effects of organizational size and availability of nearby replacement jobs.

Relationships	Coefficient	*t*-Value	*p*-Value
**Organizational size (R-sq.714)**			
Org.Emd	−0.037	−0.075	0.886
Com-Emd	−0.046	−0.173	1.821
Org.Emd_× _OS	−0.529	7.448	0.000
Com-Emd_ × _OS	−0.324	−0.726	5.22
**Availability of nearby replacement of jobs (R-sq.543)**			
Org.emd	−0.093	−0.475	3.760
Com-emd	−0.121	−0.726	0.173
Org.emb_× _NBJ	−0.669	8.753	0.000
Com-emd_ × _NBJ	−0.087	−0.338	3.118

*Org.emd, organizational embeddedness; Com.Emd, community embeddedness; OS, organizational size; NBJ, nearby replacement job.*

## Study Findings and Discussion and Implications

This study contributes to the literature of turnover theory by demonstrating robust relationships among JE, CWP, and VTOI. There are three key findings in the area of JE of young academic teaching staff.

First, this study shows that organizational-embeddedness practices significantly reduce VTOI among the young teachers, whereas community-embeddedness practices have an insignificant impact. This study extends JE-turnover research by identifying boundary conditions in terms of organizational-embeddedness and community-embeddedness to investigate the extent to which young teachers are embedded and intend to quit their jobs in the near future. Regarding particular organizational and community practices, the elements of organizational-fit and community-fit are showing a larger effect toward determining the VTOI among young teachers than other elements. Fit-related constructs appear to be the most significant; therefore, in order to control the VTOI, universities or higher academic institutions should implement policies that support the fit aspects of embeddedness.

Second, CWP plays an intermediary role which mediates the relationships between JE and VTOI. Creative role performance also affects the intention of teachers to stay in their current job and perform better. The results show that practices enhancing organizational embeddedness encourage creative role performance. The study has also found that less embedded teachers who perceive a lower level of CWP are have more intentions to leave, compared to those who perceive that they are performing well in their job. It has been found that in those universities where young teaching staffs are encouraged to perform better in their role VTOI is reduced. Also to reduce turnover in universities, the notion of embeddedness practices and CWP among young academics is supported. In order to enhance the CWP among young teachers, higher academic institutions should support their teachers by giving them the necessary resources required to perform better at the workplace.

Third, organizational size significantly moderates the relationship. If the organizational size is big (i.e., public sector university), then VTOI seems to be low independent of both organizational- and community-embeddedness. In the case of introducing community-embeddedness practices, the turnover rate in big universities is declining, whereas in small universities (i.e., private sector universities) the turnover rate is still high. This also holds for organizational embeddedness practices, but the difference is small. There might be less organizational support in private sector universities. In addition, the availability of nearby replacement of jobs also shows significant reflection in the JE and VTOI relationships but this moderation is insignificant in the case of community-embeddedness, where no interaction has been found. The turnover rate is reduced more when nearby replacement jobs are available. In the case of organizational embeddedness, a more significant moderation exists with reducing trends for both options, whereas the turnover rate is declining more when nearby replacement of jobs are not available. Public sector universities usually have their long-term embeddedness plans, whereas private sectors (i.e., small organizations) are lacking such kinds of plans; thereby it reflects other decision-criteria of staying or quitting among the young teachers. It is essential to realize such policies at the national level to give equal growth opportunities to the young teachers in small universities in the future.

### Theoretical Implications of the Study

Empirical evidences show the extrapolative validity of organizational- and community-embeddedness in the context of young teaching staff in higher academic institutions, in both the public and the private sector (Watson, 2018). Yet to our knowledge, there have been no studies so far that explored embeddedness within the context of CWP, along with the VTOI. Embeddedness is an emerging area of research and researchers are continuing the search for suitable criteria based on different classifications of embeddedness (Dechawatanapaisal, 2018a). This study provides a gateway to the studies on the effect of JE sub-dimensions on CWP and VTOI. Thus, the embeddedness criteria extracted using an appropriate framework could be applied across different other domains where organizations are facing challenges related to creative role performance and turnover. In addition, this study accentuates the importance of different JE dimensions such as link and fit in defining the CWP. Particularly, the proposed turnover theory highlighted the role performance of an employees and their JE related measures as key antecedents of VTOI in today’s organizational culture.

This study has presented CWP as an additional source of retention. The relationship found between JE and CWP suggests that teacher’s creative work has important practical implications for improving retention. Policymakers need to understand the creative role of young academics in knowledge creation and sharing. Management will need to provide adequate facilities for young teaching staff that improve embeddedness. In doing so, the teaching staff would be empowered to improve their creative performance and this would result in a higher retention rate among young academics. To develop a creative learning environment, the universities should further encourage the exchange of creative ideas among faculty members. Careful consideration should be paid to the managerial support for developing such a culture of creative work (Wang and Netemeyer, 2004; Karatepe and Vatankhah, 2014). Proven criteria of embeddedness issues of young teachers should be addressed on priority bases that could effectively reduce the turnover.

### Practical Implications of the Study

The findings of this study demonstrate different practical implications for organizations that wish to develop effective retention strategies. Specifically, this study suggests that organizations should focus on organizational-fit and community-fit constructs in their nurturing strategies to embed young teachers in their academic institutions. This study also suggests that monetary rewards only are relatively ineffective to improve retention. Hence, public and private sector universities should facilitate meaningful contributions from young teachers in creative work and provide opportunities for social interactions and personal development (Holtom et al., 2019). The findings also suggest that management should pay closer attention to the individual embeddedness strategies. A communal approach might not always work, since individual embeddedness practice is idiosyncratic in nature, and might show a different impact on employee creativity and turnover.

A series of high-performance organizational practices could enhance embeddedness, thus increasing CWP and controlling VTOI. First of all, skill upgrading is the practice of improving employees’ knowledge, skills and abilities. A combination of resources, such as training, tuition reimbursement, and socialization, can increase employee embeddedness in work, organization, or career. Second, opportunity enhancement involves individuals in the organization and their work, provides them with resources, and enables them to expand decision making power, independent team cooperation and dispute resolution. Third, encouraging and enhancing the staff’ vitality helps staff to pursue their work goals. Through the implementation of such practices, the organization can link work efforts with external compensation, thereby improving on-the-job and off-the-job embeddedness.

Similarly, in the case of community-embeddedness practice, nearby extended families can form a valuable support network to help employees (or their immediate relatives) in managing the conflict between work and family needs. The value of this network depends on the strength of family relationships, how many large families are nearby, and whether employees view these relationships positively. Employees and their immediate family members live in communities that provide a variety of benefits and attributes. Communities that offer multiple resources (for example, good schools, universities, public transport) are more likely to embed employees and their immediate family locally by better meeting their specific needs.

Finally, Professional communities are pervasive within the increasingly “boundary less” career environment. They encourage occupational embeddedness by offering chances for employees to gain specialized knowledge, network with others, and present new research and practices.

## Conclusion

University staffs are considered to be the most valuable assets in education and research. When an experienced and competent teacher shows an intention to leave his/her current job, productivity and academic performance decrease. This study explores the different dimensions of JE like organizational- and community-embeddedness, which play an important role in improving work performance and reducing the turnover rate of young teachers. In this study, a comprehensive questionnaire was designed for the proposed model and the data were collected through the mail-survey process. Young teaching staffs in different private and public sectors higher academic institutions (i.e., universities) were asked to record their responses. In order to test the model, variance-based structural equation model is applied to test the hypotheses. Direct, indirect and total effects are generated to evaluate the path model.

The link/fit–dimensions of organizational- and community-embeddedness are more critical in determining the relationships with CWP and VTOI. The study suggests that organizations should focus on organizational-fit and community-fit constructs in their nurturing strategies to embed young teachers in their academic institutions in the future. Organizational practices, such as skill-enhancing, opportunity-enhancing, and motivation-enhancing practices might support teachers’ embeddedness. The two aspects of the link-dimension of embeddedness and the encouragement of creative work performance could be employed to embed teachers into the organization. This paper contributes to the existing literature by providing exploratory answers to the challenging questions put forward at the beginning: “what can make young teachers perform and stay?” Embeddedness related decision criteria are of crucial importance if universities wish to find appropriate ways to maximize teachers’ performance and retention.

### Limitations and Future Research

The findings of this research should be viewed in the light of certain limitations in research design, methodology, and model development of the study. This study used the sample from comparatively large and mostly public sector universities, however, future research might include the sample from different organizations in services sector to check the generalizability of the findings of this study. It is highly important to explore the extended version of JE constructs as suggested by Peltokorpi et al. (2017). Future research may be extended to examine JE, CWP, and TOI through qualitative research to draw more rich and varied information and opinion. The literature regarding JE, CWP, and TOI provided evidence of linear relationships between all variables. However, in future longitudinal research projects may be carried out to find out the relationship and effects of study variables in academic setting.

As a relatively new theory of turnover research, JE involves the relationship with other organizational behavior structures and staff turnover. In-depth study of this relationship can not only enrich JE theory but also improve the existing turnover models as JE and VTOI are affected by a number of other organizational factors (Peltokorpi et al., 2017; Shehawy et al., 2018). Future research may reveal other aspects and moderating factors that affect this relationship, such as demographic characteristics at the individual level (e.g., career stage, work level, work generation); personal tendencies; and work characteristics. This study considered two possible moderators in the measurement model. In addition, the study can be expanded to find the influence of other potential moderating factors, such as commuting time, financial requirements and working group cohesion as recommended by Zhang et al. (2012) and Coetzer et al. (2019).

In recent studies, organizational justice has appeared to be an important aspect of JE among employees having job burnout issues in organizations (Holtom et al., 2019). Therefore, the future research can include the entire justice scale to capture the JE level of the employees along with the mediating role of job burnout to measure the retention rate.

## Data Availability Statement

The raw data supporting the conclusions of this article will be made available by the authors, without undue reservation.

## Ethics Statement

Ethical review and approval was not required for the study on human participants in accordance with the local legislation and institutional requirements. Written informed consent for participation was not required for this study in accordance with the national legislation and the institutional requirements.

## Author Contributions

IS conceived of the presented idea and developed the theory and performed the computations. SS and FA verified the analytical methods. AY encouraged to investigate and supervised the findings of this work. All authors discussed the results and contributed to the final manuscript. IS carried out the experiment and wrote the manuscript with support from fabricated the BS sample. AY helped to supervise the project.

## Conflict of Interest

The authors declare that the research was conducted in the absence of any commercial or financial relationships that could be construed as a potential conflict of interest.

## Annexure

**TABLE A1:** Scale for measuring the Job Embeddedness among young teachers.

**Fit-community** 1. I love the place where I live. 2. The weather where I live is suitable for me. 3. This community is a good match for me. 4. I think of the community where I live like home. 5. The area where I live offers the leisure activities that I like.
**Fit-organization** 1. I like the members of my workgroup. 2. My co-workers are similar to me. 3. My job utilizes my skills and talents well. 4. I feel like I am a good match for this company. 5. My values are compatible with the organization’s values. 6. I fit with the company’s culture. 7. I like the responsibility and authority I have at this company. 8. I can reach my professional goals working for this organization. 9. I feel good about my professional growth and development.
**Links-organization** 1. How long have you been in your present position? 2. How long have you worked for this company? 3. How long have you worked in this industry? 4. How many co-workers do you interact with regularly? 5. How many co-workers are highly dependent on you? 6. How many teams are you on? 7. How many committees are you on?
**Links-community** 1. Are you currently married? 2. If you are married, does your spouse work outside the home? 3. Do you own the home you live in? 4. My family roots are in this community. 5. How many family members live nearby? 6. How many of your close friends live nearby?
**Sacrifice-organization** 1. I have a lot of freedom on this job to decide how to pursue my goals. 2. The perks on this job are outstanding. 3. I feel that people at work respect me a great deal. 4. I would sacrifice a lot if I left this job. 5. My promotional opportunities are excellent here. 6. I am well compensated for my level of performance. 7. The benefits are good at this job. 8. The health care benefits provided by this organization are excellent. 9. The retirement benefits offered by this organization are excellent. 10. The prospects for continuing employment with this company are excellent.
**Sacrifice-community** 1. Leaving this community would be very hard. 2. People respect me a lot in my community. 3. My neighborhood is safe.
**Creative work performance** 1. Delivering lectures in innovative ways. 2. Carrying out office tasks in ways that are creative resourceful. 3. Improvising methods for solving a problem when an answer is not apparent. 4. Generate and evaluate new solutions for old problems.
**Voluntary turnover intention** 1. I would like to leave my present employer. 2. I plan to leave my current employer as soon as possible. 3. Under no circumstances will I leave my voluntary leave my present employer.
